# Novel Handheld Device for Remote Monitoring of Dry Macular Degeneration and Patient Usability Assessment

**DOI:** 10.3390/diagnostics15111353

**Published:** 2025-05-28

**Authors:** Angela C. Yim, Lyna Azzouz, Yannis M. Paulus

**Affiliations:** 1 Department of Ophthalmology and Visual Sciences, University of Michigan, Ann Arbor, MI 48105, USA; ayim@med.umich.edu (A.C.Y.); lazzouz@stanford.edu (L.A.); 2 Department of Ophthalmology, Stanford University, Stanford, CA 94305, USA; 3 Department of Ophthalmology, Wilmer Eye Institute, Johns Hopkins University, Baltimore, MD 21287, USA; 4 Department of Biomedical Engineering, Johns Hopkins University, Baltimore, MD 21287, USA

**Keywords:** optical monitoring device, age-related macular degeneration, AMD, device usability assessment, remote monitoring device, shape discrimination hyperacuity

## Abstract

**Background/Objectives:** A novel, handheld, standalone device using shape discrimination hyperacuity has been developed to remotely monitor age-related macular degeneration (AMD). **Methods:** We clinically validated the device in an outpatient dry AMD population to evaluate its usability and comfort. A cross-sectional study was conducted with subjects aged 50 years or older with dry AMD at the University of Michigan Kellogg Eye Center outpatient clinic after approval from the UM IRB (HUM00187177). Subjects used the device and then completed a device survey and System Usability Scale (SUS). **Results:** Thirty-one subjects completed the study, and one subject withdrew post-study completion (mean age 77 years, STD 8 years). The mean testing time was 126 s (STD 56 s), and the median was 116 s. Most patients reported that the use of the device occurred for an acceptable duration (97%), was easy (97%), and was comfortable (90%). The mean SUS score was 77.7 (STD 11.9). **Conclusions:** A handheld, standalone device can provide a rapid, easy, and comfortable testing solution for patients with dry AMD. The usability of the device supports further clinical trials to demonstrate the device’s ability to reliably detect the progression of AMD.

## 1. Introduction

Age-related macular degeneration (AMD) is the leading cause of irreversible vision loss in American adults aged 50 years or older [1,2]. As a degenerative disease of the retina, AMD causes progressive damage to the macula, leading to central vision loss that can progress to blindness. The early stage of AMD (dry AMD) is characterized by lipid and protein deposits called drusen and tends to be asymptomatic [3]. However, 13% of patients with dry AMD will develop the advanced stage or wet AMD within 5 years [4]. Wet AMD involves the proliferation of—and damage from—blood vessels in the subretinal space, which can cause irreversible damage and vision loss [5].

Timely intervention with anti-vascular endothelial growth factor treatment within 1–2 weeks of the transition from dry to wet AMD has been shown to prevent further vision loss and blindness [6,7]. However, the current standard of care involves patients with dry AMD receiving periodic monitoring every 6–12 months at their ophthalmologist’s office with examinations and imaging (e.g., optical coherence tomography [OCT]) [8]. In between office visits, patients can self-monitor using the Amsler grid, which was developed in the 1940s. Despite its ease of use and low cost, the Amsler grid has been reported to have high false negative rates in detecting both early and late AMD [9,10]. The limitations of home monitoring with the Amsler grid and infrequent office visits can lead to a gap in the timely identification of AMD transition, hindering the administration of treatment during the optimal time window. Thus, there is a need to develop an affordable, remote monitoring solution for AMD to prevent vision loss.

Several products have recently been developed to remotely monitor AMD progression. In 2009, ForeseeHome (Notal Vision, Ltd., Tel Aviv, Israel) received clearance from the U.S. Food and Drug Administration (FDA) for home use by patients with dry intermediate AMD and a best corrected visual acuity of 20/60 or better [11]. ForeseeHome is a standalone device based on hyperacuity that requires patients to respond to visual stimuli displayed on a screen. The randomized, controlled HOME clinical trial found that participants who used the monitoring device had better visual acuity at the time of detection of AMD-associated choroidal neovascularization than the control group who used standard care, including the Amsler grid [12]. However, the 10-year retrospective ALOFT study found that 29% of patients received a false positive alert of AMD progression from the device [13].

Two home-based OCT prototypes (Notal Vision Home OCT and SELFF-OCT) have been developed with favorable usability data. However, challenges include capturing high quality images and utilization of the device during travel along with significant costs associated with OCT [14,15]. With the rise of smartphone use, seven phone-based applications are currently available for monitoring AMD [16]. Of the four applications cleared by the FDA, Home Vision Monitor (Roche-Genentech, San Francisco, CA, USA) also uses shape discrimination hyperacuity and has been shown to differentiate between normal, early, and intermediate AMD [17]. However, this application faces usability challenges with the AMD population as installation of the app and first use of the app was negatively associated with age, which is a risk factor for AMD progression [18]. Shape discrimination hyperacuity is the visual ability to detect subtle changes or small distortions in the shape of a visual stimulus, such as a circle. It is considered a form of hyperacuity because it can detect changes smaller than those detectable by traditional visual acuity tests.

To fulfill this clinical need for an easy-to-use, portable device for home monitoring to rapidly screen patients with dry AMD for wet AMD, we developed a device using shape discrimination hyperacuity to identify patients’ minimum distortion-detection threshold (MDDT). To determine the usability of this device, we clinically validated it with dry AMD patients. The goal of the study was to evaluate the length of time needed to use the device as well as its usability and comfort. Qualitative feedback was gathered to improve future generations of the prototype.

## 2. Materials and Methods

A cross-sectional study was conducted with subjects aged 50 years or older with dry AMD at the University of Michigan Kellogg Eye Center outpatient retina clinic after approval from the UM Institutional Review Board (IRB) (HUM00187177, PI: Y.M. Paulus). The study was registered on clinicaltrials.gov (NCT06178978). Eligible patients were identified by review of clinic schedules, according to the following criteria: 50 years or older with at least one eye with dry AMD. Major exclusion criteria included retinal pathologies other than dry AMD. Informed consent was obtained, and the study adhered to the tenets of the Declaration of Helsinki. During a one-month period in March 2022, 31 participants enrolled in and completed the study, one of whom withdrew post-study completion.

Participants received a brief (<5 min) standardized tutorial on how to use the device. They used the device on the eye with dry AMD, and the test duration was recorded. If both eyes had dry AMD, participants tested both eyes in succession. The study only evaluated eyes with dry AMD. Afterwards, they completed a device survey with eight questions using a Likert scale and an optional fill-in question for suggestions to improve the device. Participants then filled out the standardized System Usability Scale (SUS) survey that also uses a Likert rating scale ranging from 1 (strongly agree) to five (strongly disagree) for 10 statements. The SUS survey was developed in 1996 by John Brooke and has been used as a reliable method for collecting subjective user device experiences [19].

### Statistical Methods

All statistical analyses were performed using Microsoft Excel 2021 software (Microsoft Corporation, WA, USA). The findings are expressed as mean ± standard deviation (SD). Statistical significance was defined as a *p*-value of less than 0.05. The *p*-value was calculated for test duration with the two-sided Wilcoxon signed rank test for paired data using the online calculator https://astatsa.com/WilcoxonTest/ (accessed on 20 May 2025). All *p*-values are reported in the results, and significant values are highlighted in bold.

The SUS Likert scores were converted to an overall SUS score using the standard method by multiplying the sum of the Likert scores by 2.5 [20]. The SUS score is a percentile score on a range of 0–100, and scores above 68 are believed to be above the global average [21].

## 3. Results

### 3.1. Device

**Figure 1:**
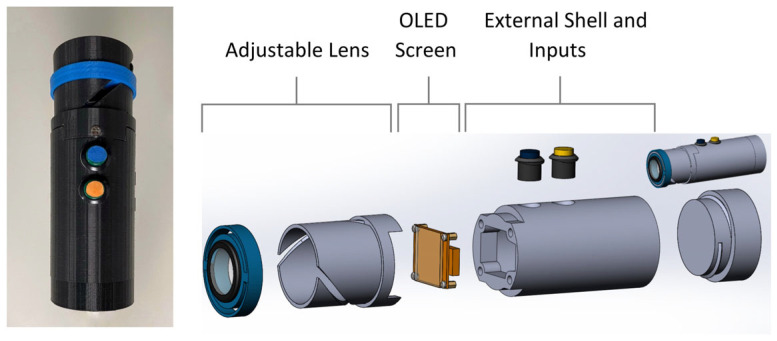
Left. External photograph of the device demonstrating the 3D-printed shell and 2 buttons. Right. Exploded view of hardware components.

**Figure 2:**
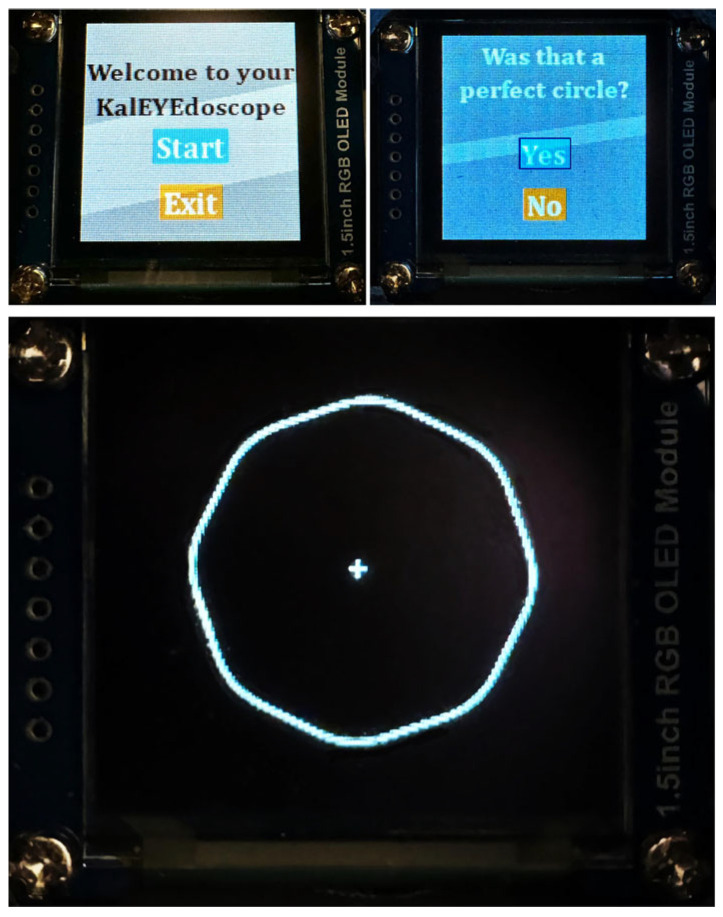
The graphic user interface images appearing on the device’s screen with user prompts (top), along with a circle-like image (prompt), which patients are asked to evaluate for perfect circularity.

A novel, digital, handheld, standalone device was developed using shape discrimination hyperacuity to identify patients’ MDDT to monitor the conversion of dry to wet AMD. The prototype device comprises an injection-molded 3D-printed shell, two tactile buttons, a battery, a processing unit (Raspberry Pi 3), and a 1.5-inch RGB OLED screen (Figure 1). It weighs 0.21 kg and has a diameter of 61 mm. The power supply is a rechargeable Adafruit USB Battery Pack for Raspberry Pi, which can power a Raspberry Pi for up to 15 h. Given the mean test duration of 126 s, this could power up to 428 tests after a single charge, although this was not tested in this study. Patients place the monocular device proximal to their eye and adjust the lens until the screen is in focus. They are presented with a series of circle-like images along with a prompt asking whether the image previously displayed was a perfect circle (Figure 2). They respond to the prompts by pushing the appropriate button, and their responses are collected and analyzed longitudinally to determine changes in their MDDT. The push buttons were activated with a resistance force of 1.52 N, which was between the 1.4 and 5.6 N desired force to ensure it was easy to push.

### 3.2. Patient Characteristics

All 31 participants completed the study without issue. One participant withdrew post-test completion. Participant characteristics are included in Table 1. Participants had a mean age of 77.4 years with a standard deviation of 7.9 years. Participant gender was 50% male and 50% female. Racial and ethnic data retrospectively collected from subjects’ electronic medical records revealed that 100% and 90% of participants self-reported as white and non-Hispanic, respectively. Of the 30 participants, 35 eyes were tested.

### 3.3. Testing Duration

The mean testing duration was 126 s ± 57 s with a range of 46 to 243 s. The median testing duration was 116 s. The test was performed in the clinic under the supervision of a study coordinator. All patients had dry AMD, but five participants had dry AMD in both eyes and used the device twice, once in each dry AMD eye. All five patients completed testing of the second eye in a shorter amount of time than the first eye. However, there was no statistically significant difference between the first and second eye test durations on the two-sided Wilcoxon signed rank exact test (*p* = 0.065). Given the small sample size of only five patients with both eyes tested and the minimum sample size of six required to achieve a *p* < 0.05 with the Wilcoxon signed rank exact test, the small sample size precludes the ability to achieve statistical significance. The mean test duration of the first eye was 128 s compared to the second test duration of 84 s.

### 3.4. Device and SUS Surveys Results

Of the 30 participants, 29 (97%) reported that the time required to complete the test was acceptable (Table 2 and Table 3). The one participant who reported dissatisfaction with the testing duration completed the test in 111 s, which is faster than the mean duration of 126 s.

The comfort of using the device was assessed with three questions on the device survey. Ninety percent of the participants reported that the device was comfortable to hold. One participant indicated that the device was bulky and required the use of both hands. Some participants used both hands to operate the device, but did not voice or write down concerns about this. 100% of participants agreed that the device had an acceptable weight and that its two buttons were easy to push. Eighty-seven percent did not report that the device was cumbersome or awkward. Two participants gave similar recommendations to improve the comfort of the device by adding cushioning to the outer rim of the eyepiece.

The device survey also assessed participants’ acceptance of the length of time required for individual portions of the test. All participants found the time required for the device to turn on and be ready for use acceptable. Of the participants, 83% and 77% reported that the time for the circle to appear and the time to screen transition were acceptable, respectively. The length of time the circle appeared on the screen was perceived to be too short by 13% of participants.

Almost all participants (97%) reported that learning how to use the device was easy, and 100% indicated they believed most people would learn to use it quickly. Only 7% of participants reported they needed to learn a lot of things before using the device. The participants’ mean SUS score was 77.7 (SD 11.9), which suggests the device’s usability is above the average usability of all devices.

## 4. Discussion

This is the first description and clinical evaluation of a handheld, standalone device that can provide a rapid, easy, and comfortable testing solution for patients with dry AMD. A majority of patients found it easy to learn how to use the device without the need for support from a technical expert.

This device was designed for a target user population of elderly patients with dry AMD. Efforts were made to minimize the number and complexity of user actions required to start and complete the test, which may positively contribute to the testing duration. Aside from plugging the device into its battery source, the user only needs to push one of two buttons to use the device. Each button was designed to be operable with minimal force to be easy to use by older populations, since hand grip and pinch strength have been shown to decrease with age [22,23]. Our intentions to avoid user muscle fatigue through a lightweight device and manageable circumference were aligned with the results of participant satisfaction with these features.

The device’s usability was well accepted by participants, although two improvements were suggested. First, the eyepiece, which is part of the 3D-printed plastic shell, could be more comfortable with additional padding. Second, the length of time the circle is displayed was too short for a few participants. Future iterations of the device will incorporate participant feedback to include a padded eyepiece and lengthen or customize the circle appearance duration. It is unknown how the increased or personalized circle duration will impact overall testing time.

While there are other AMD home-monitoring products on the market, this device is uniquely monocular, which eliminates the filling-in bias found with binocular testing devices [24]. This filling-in mechanism occurs in AMD patients when they fill in the scotoma through peripheral patterns provided by binocular vision [25]. Furthermore, this device is a standalone device and does not rely on smartphone or tablet technology, which is utilized by alternative products, and it is compact and easy to use. Although studies have shown that 80% of visually impaired adults use smartphones, there is a lack of evidence of the usability of mobile apps for the elderly [26,27]. Studies have evaluated shape discrimination hyperacuity and demonstrated its utility in monitoring AMD and detecting the progression from dry to wet AMD [28,29,30,31].

The results of this study demonstrate that testing duration is an advantage of this device. Its mean test duration of 126 s and median test duration of 116 s is a 41% decrease in testing time compared to another AMD home-monitoring product, the ForeseeHome [11]. Furthermore, there is potential for a decrease in testing duration as patients become more familiar with the device. The small subset of participants who completed testing on two eyes completed the second test faster at an almost statistically significant level of 84 s, which was a 34.4% reduction in test duration after only a single use. Given that home monitoring would be completed on a regular basis, we anticipate that, in clinical use, patients would become quite facile at using the device and could thus reduce their testing duration. In addition, 16% of patients prescribed the ForeseeHome device were unable to use it, whereas all patients in this study were able to use the study device [32].

A major strength of this study is that it is the first description of this novel device and is a prospective design for the target user population of elderly patients with dry AMD. This study assesses the target patient population’s perception of device usability.

This study has several limitations. It was conducted at one outpatient retina clinic with a relatively small, homogeneous sample, as this patient population was 100% Caucasian and 90% non-Hispanic, which limits the generalizability of the findings to more diverse populations. Furthermore, this study was limited to patients with dry AMD and excluded other retinal pathologies, and thus it is unclear how this device will perform in patients with multiple retinal pathologies, varying severities of macular degeneration, or with more variable visual acuities. Finally, this study administered a device-specific survey that has not been previously tested or validated. However, the survey’s statements are simple and straightforward, with minimal interpretation needed.

The usability of the device supports further larger-scale clinical trials to demonstrate its ability to reliably detect the progression of AMD. Accurate detection of AMD progression with the device could enable more timely treatment intervention to improve patient outcomes and improve long-term quality of life through improved visual outcomes. Thus, further research investigating the device’s reliability and patient adherence to improve the outcomes of patients with AMD is warranted.

## Figures and Tables

**Table 1:** Study population patient characteristics *.

Characteristic	Value
Age (years), mean (SD)	77.4 (7.9)
Age Group	
50–64 years of age (%)	3 (10.0)
65–74 years of age (%)	8 (26.7)
75–84 years of age (%)	13 (43.3)
85+ years of age (%)	6 (20.0)
Female gender (%)	15 (50)
Race *	
White	30 (100%)
Ethnicity	
Non-Hispanic	27 (90%)
Unknown	3 (10%)
Eyes	35
Right eye (%)	20 (57.1)
Left eye (%)	15 (42.9)
Both eyes (%)	5 (14.3)

* Data represents number of participants unless otherwise indicated. SD = standard deviation.

**Table 2:** Summary of results from the device survey and System Usability Survey *.

Statement Number	Statement	Mean Likert Score *, (SD)
DS 1	The device was comfortable to hold for the duration of the test.	4.4 (0.9)
DS 2	The device weight was acceptable.	4.8 (0.4)
DS 3	The buttons were easy to push.	4.9 (0.3)
DS 4	I found it easy to learn how to use the device.	4.7 (0.7)
DS 5	The time it took to complete the test was acceptable.	4.8 (0.8)
DS 6	The time it took for the screen to transition was acceptable.	4.2 (1.3)
DS 7	The time it took for the circle to appear on the screen was acceptable.	4.4 (0.9)
DS 8	The time it took for the device to turn on and be ready for use was acceptable.	4.9 (0.3)
SUS 1	I think that I would like to use the device frequently.	3.6 (1.2)
SUS 2	I found the device unnecessarily complex.	1.3 (0.6)
SUS 3	I thought the device was easy to use.	4.8 (0.6)
SUS 4	I think that I would need the support of a technical person to be able to use the device.	1.2 (0.4)
SUS 5	I found the various functions in the device were well integrated.	4.5 (0.8)
SUS 6	I thought there was too much inconsistency in the device.	1.5 (0.9)
SUS 7	I would imagine that most people would learn to use the device very quickly	4.9 (0.3)
SUS 8	I found the device very cumbersome (awkward) to use.	1.5 (1.1)
SUS 9	I felt very confident using the device.	4.0 (1.2)
SUS 10	I needed to learn a lot of things before I could get going with the device.	1.4 (0.8)

* Participants rated their perspective of each statement on the following Likert scale: 1 = strongly disagree, 2 = disagree, 3 = neutral, 4 = agree, and 5 = strongly agree. DS = device survey.

**Table 3:** Individual participant device survey and System Usability Survey results ^1^.

Statement			Participant																											
Number ^1^	1	2	3	4	5	6	7	9	10	11	12	13	14	15	16	17	18	19	20	21	22	23	24	25	26	27	28	29	30	31
DS 1	1	5	4	3	5	5	4	4	5	5	5	4	4	5	5	5	5	5	4	5	5	3	5	5	4	4	4	5	5	5
DS 2	4	5	5	5	5	5	4	5	5	5	5	5	5	5	5	5	5	4	4	5	5	5	5	5	5	5	4	5	5	5
DS 3	4	5	5	5	5	5	5	5	5	5	5	5	5	5	5	5	5	5	4	5	5	5	5	5	5	5	4	5	5	5
DS 4	2	5	3	5	4	5	5	5	5	5	5	5	5	5	5	5	5	4	4	5	5	5	5	5	5	4	4	5	5	5
DS 5	5	5	4	5	5	5	1	5	5	5	5	5	5	5	5	5	5	5	5	5	5	5	5	5	5	4	4	5	5	5
DS 6	5	2	2	5	5	5	5	5	5	5	5	5	3	2	5	4	5	3	5	5	5	2	4	1	5	5	4	5	5	5
DS 7	4	4	4	5	4	5	5	3	5	5	3	5	2	5	5	5	5	3	5	5	5	2	4	5	5	4	4	5	5	5
DS 8	4	5	4	5	4	5	5	5	5	5	5	5	5	5	5	5	5	5	5	5	5	5	5	5	5	5	4	5	5	5
SUS 1	2	2	4	5	2	2	2	4	5	4	4	4	4	2	5	5	5	4	3	2	5	5	3	5	2	4	3	5	5	2
SUS 2	1	3	1	1	1	3	2	1	1	1	2	1	1	1	1	2	1	1	1	1	1	2	1	1	1	1	2	1	1	1
SUS 3	4	2	5	5	5	5	4	5	5	5	5	5	5	5	5	5	5	5	5	5	5	5	5	5	5	5	4	5	5	5
SUS 4	1	1	2	1	1	1	1	1	1	1	1	1	1	2	2	1	1	1	1	1	1	1	1	1	2	1	2	1	1	1
SUS 5	4	4	5	5	4	2	5	5	5	5	4	5	5	3	5	5	5	5	5	3	5	4	5	5	5	3	4	5	5	5
SUS 6	2	4	1	1	1	2	2	1	1	4	1	1	1	2	1	1	1	1	2	1	1	3	1	1	1	1	2	1	1	1
SUS 7	4	5	5	5	5	4	5	5	5	5	5	5	5	5	5	5	5	5	5	5	5	5	5	5	5	5	4	5	5	5
SUS 8	5	1	1	1	1	2	3	1	1	1	1	2	1	2	1	1	1	4	1	1	1	1	1	2	1	1	4	1	1	1
SUS 9	1	2	5	5	2	5	5	5	4	3	4	5	3	2	5	5	5	4	5	3	5	3	5	2	4	4	4	5	5	5
SUS 10	4	1	4	1	1	1	2	1	1	1	1	1	1	2	1	1	1	1	2	1	1	1	1	1	1	2	2	1	1	1

^1^ Participants rated their perspective of each statement on the following Likert scale: 1 = strongly disagree, 2 = disagree, 3 = neutral, 4 = agree, and 5 = strongly agree. DS = device survey. SUS = system usability survey.

## Data Availability

The de-identified, anonymized datasets used during the current study are available from the corresponding author on reasonable request.

## Abbreviations

The following abbreviations are used in this manuscript:

AMD	age-related macular degeneration
UM	University of Michigan
IRB	Institutional Review Board
SUS	System Usability Scale
STD	Standard deviation
OCT	optical coherence tomography
FDA	United States Food and Drug Administration
MDDT	minimum distortion-detection threshold
RGB	Red green blue
OLED	Organic light-emitting diode
SD	Standard deviation
